# Prevalence and geo-clinicodemographic factors associated with hepatitis B vaccination among healthcare workers in five developing countries

**DOI:** 10.1186/s12879-022-07556-3

**Published:** 2022-07-07

**Authors:** Precious Adade Duodu, Ernest Darkwah, Pascal Agbadi, Henry Ofori Duah, Jerry John Nutor

**Affiliations:** 1 Department of Nursing and Midwifery, School of Human and Health Sciences, University of Huddersfield, Queensgate, Huddersfield, England, UK; 2 Department of Psychology, University of Ghana, P.O. Box LG 84, Legon, Ghana; 3 Department of Sociology and Social Policy, Lingnan University, Tuen Mun, Hong Kong, SAR China; 4 Research Department, FOCOS Orthopaedic Hospital, Accra, Ghana; 5 Department of Family Health Care Nursing, School of Nursing, University of California, San Francisco, CA USA

**Keywords:** Prevalence, Hepatitis B vaccination, Healthcare workers

## Abstract

**Background:**

There is a four-fold risk for hepatitis B infection among healthcare workers compared to the general population. Due to limited access to diagnosis and treatment of hepatitis B in many resource-constrained settings, there is a real risk that only few healthcare workers with viral hepatitis may get screened or diagnosed and treated. Studies on hepatitis B vaccination among healthcare workers in developing countries are sparse and this bodes ill for intervention and support. The aim of the study was to estimate the prevalence and explored the associated factors that predicted the uptake of the required, full dosage of hepatitis B vaccination among healthcare workers (HCWs) in five developing countries using nationally representative data.

**Methods:**

We used recent datasets from the Demographic and Health Surveys Program’s Service Provision Assessment Survey. Descriptive summary statistics and logistic regressions were used to produce the results. Statistical significance was pegged at p < 0.05.

**Results:**

The proportion of HCWs who received the required doses of hepatitis B vaccine in Afghanistan, Haiti, Malawi, Nepal, and Senegal were 69.1%, 11.3%, 15.4%, 46.5%, and 17.6%, respectively. Gender, occupational qualification, and years of education were significant correlates of receiving the required doses of hepatitis B among HCWs.

**Conclusions:**

Given the increased risk of hepatitis B infection among healthcare workers, policymakers in developing countries should intensify education campaigns among HCWs and, perhaps, must take it a step further by making hepatitis B vaccination compulsory and a key requirement for employment, especially among those workers who regularly encounter bodily fluids of patients.

## Background

Over the past three decades, significant global health efforts have been made to control viral hepatitis [1, 2], including initiatives on blood safety, healthcare injection safety, infection control, harm reduction for intravenous drug users, and vaccination [3, 4]. There have been global policies by the World Health Organization (WHO) such as the Global Hepatitis Programmes (2011 and 2014) to improve public awareness and drastically reduce hepatitis infections [4]. Also, there was the 2016–2021 Global Health Sector Strategy on viral hepatitis in 2016 which aims to eliminate viral hepatitis by 2030 by reducing new infections by 90% and mortality by 65% [4, 5]. Despite these significant efforts, hepatitis B virus (HBV) infection continues as a serious public health concern [6, 7]. Globally, an estimated 237 million people live with chronic HBV with about 900,000 deaths [8]. The majority of the disease burden is in WHO African and Western Pacific Regions, affecting 6.1% and 6.2% of the adult populations respectively [5, 9]. Although preventable, HBV is transmitted mostly through mother-to-child during birth and delivery (vertical transmission), percutaneously or mucosal exposure to infected bodily fluids such as sexual intercourse with an infected partner, injection-drug use, needle injuries, and occupation-risk healthcare workers [HCWs] [10, 11].

Worldwide, millions of healthcare professionals work in healthcare institutions [10]. Due to the nature of their work, HCWs are more prone to HBV infections. There is a four-fold risk for HBV infection among HCWs compared to the general population [12, 13]. Studies in sub-Saharan Africa indicate that more than half a million of HCWs are infected with HBV infections annually [10, 11]. With over 5% of healthcare-related injections being unsafe [4], there is about 30% risk of getting HBV infection from a single needlestick injury compared to hepatitis C virus (3%) and HIV (0.3%) [11, 14]. The most effective and feasible means of preventing HBV infection are by avoiding exposure to infected bodily fluids, screening, and vaccination [3, 14, 15]. Screening links people to interventions to reduce transmission through risk behaviour counselling and hepatitis B vaccination. However, limited access to hepatitis B diagnosis and treatment in resource-constrained settings is challenging [7].

In 1982, a safe and effective vaccine that offers 90–100% protection against the HBV was discovered [16, 17]. Following this, hepatitis B vaccination continues to be a part of the WHO Extended Programme for Immunization [18]. This underlines the need for HCWs who are exposed to the HBV infection on daily bases to be vaccinated against the disease. Complete vaccination against HBV is achieved by a three-dose regimen administration, with the second and third doses given at 1 and 6 months after the initial dose, following which a test for hepatitis B surface antibody should be carried out 6–8 weeks after the final dose [14, 16]. In the event of low antibody levels, a booster dose should be given immediately to improve response, followed by a blood test in 6–8 weeks to check the response to the booster dose [14, 19]. According to the Centers for Disease Control and Prevention (CDC), all HCWs should receive the 3-dose vaccine regimen with an approximate protection rate of 30–55% after the first dose, 75% after the second dose, and > 90% after the third dose in adults aged ≤ 40 years [3, 20]. However, adherence to vaccinations in general is said to be poor among HCWs, especially in developing countries [11, 13, 21]. The estimated hepatitis B vaccination rate among HCWs range from 18 to 39% in developing countries compared to 67–79% for their counterparts in developed countries [10, 11, 22]. Previous studies have reported the type of profession, years of education, years of working experience, and gender among others as predictors of low hepatitis B vaccination uptake among HCWs [3, 10, 11, 23, 24].

Despite the critical nature of HBV infections among HCWs, there is paucity of literature on the uptake of hepatitis B vaccinations among HCWs in endemic countries such as Afghanistan, Haiti, Malawi, Nepal, and Senegal. Although data on specific endemicity of HBV in these countries is limited, the available statistics show an overall prevalence rate of 6.15%, 6.54%, 1.53% and 1.76% among injecting drug users, female sex workers, obstetric patients and blood donors respectively in Afghanistan alone [25, 26]. Also, Haiti is reported to be the only country in the Antimicrobial Resistance (AMR) that is highly endemic in HBV with a reported HBsAg prevalence > 7% [27]. Systematic reviews have reported a pooled prevalence of 8.1% among adults in Malawi and chronic HBV prevalence of ≥ 11% for Senegal with an estimated 85% of the Senegalese population reported to have experienced HBV infection [28, 29]. Senegal is therefore described as a hyperendemic country with regards to HBV. In Nepal, although the overall prevalence rate is reported to be low, a high prevalence rate (≥ 8%) has been reported by various studies among sub-groups within the population especially in the mountain regions [30].

A combined hepatitis B vaccine given at 6, 10, and 14 weeks of age as part of National Immunization Schedule were introduced in Nepal, Senegal, Malawi, Afghanistan, and Haiti in 2002 [31, 32], 2002 [33], 2004 [34], 2006 [35], and 2012 [36, 37] respectively. However, the birth dose was introduced only in 2014 in Afghanistan [35], while Senegal introduced the monovalent birth-dose vaccine in 2016 [34]. Yet, the Senegalese government lacks vaccination programme for high risk population such as HCWs [31, 32]. Contrary to the WHO recommendations, a birth dose of hepatitis B vaccine is not included or implemented in the present vaccination schedule in Haiti [36] and Malawi [33]. Although immunization of HCWs before they come into contact with patients at all levels is recommended by the Malawian Ministry of Health, there is inconsistent adherence to and enforcement of such recommendations [38]. The existing data provides an overview of overall prevalence without providing insights into specific important sub-groups such as health worker groups. Therefore, this study estimated the prevalence and explored the associated factors that predicted the uptake of the required, full dosage of hepatitis B vaccination among HCWs in these five developing countries using nationally representative data.

## Methods

### Study design and data source

In all, there were 13 developing countries who participated in the Service Provision Assessment (SPA) surveys under the Demographic and Health Survey (DHS) program. Only formal facilities are included in the SPA survey hence pharmacies and individual doctors’ offices are often excluded from the SPA. For the purposes of our study, we selected countries with recently conducted SPA surveys from 2013 up till now and having questions on hepatitis B screening for HCWs. The countries that met the criteria were Afghanistan (2018–2019), Haiti (2017–2018), Malawi (2013–2014), Nepal (2015), and Senegal (2019), and these SPA surveys were conducted by the countries’ respective national agencies with technical support from the Demographic and Health Surveys (DHS). Besides the standard DHS, the Service Provision Assessment survey is undertaken to evaluate the health system readiness to provide quality care, amongst others. The surveys used generic questionnaires to assess the availability of essential services and assessed the presence of standard facilities and equipment required to provide these essential services. These services included child welfare clinics, and immediate newborn care, maternal and child health, antenatal services, birth control, treatment for sexuality transmitted diseases, tuberculosis, malaria, and non-communicable diseases amongst other essential health care services. These generic questionnaires included “facility inventory questionnaire”, “health provider interview questionnaire”, “observation protocols for antenatal care, family planning, services for sick children, and normal obstetric delivery and immediate newborn care” and “exit Interview questionnaires for antenatal care and family planning clients and for caretakers of sick children whose consultations were observed”. The survey also assessed health system readiness to ensure infection prevention in the health environment. The healthcare facilities included governmental, private, and quasi-governmental facilities in both rural and urban areas. Along with data on health system preparedness, data on vaccination history of health care providers were obtained. The unit of measurement included both the health facility and individual health care providers.

### Data collection and sample size

Computer-Assisted Personal Interviews (CAPI) were used to collect data from HCWs who were working in sampled healthcare facilities. For Afghanistan, the 1038 HCWs selected were all interviewed, representing 100% response rate [39]. For Haiti, 4461 HCWs were interviewed out of 4680; this represents 95.32% response rate [40]. For Malawi, the selected 2660.0 HCWs were all interviewed, representing 100% response rate [41]. For Nepal, 4057 HCWs were interviewed out of the selected sample of 4216; this represents 96.23% response rate [42]. For Senegal, 1353 HCWs were interviewed out of 1355; this represents 99.99% response rate [43]. The following demographic characteristics of the HCWs were collected: years of education, sex, and qualification. The following were the weighted analytic sample sizes for each of the countries included in the study: Afghanistan, N = 1038.0; Haiti, N = 4461.0; Malawi, N = 2660.0; Nepal, N = 4057.0; Senegal, N = 1353.0. There were no missing cases in the datasets.

### Outcome and explanatory variables

The outcome variable is complete dosing of hepatitis B vaccination among healthcare providers. The HCWs were asked the following ‘Yes/No’ question to know their hepatitis B vaccination status: “Have you received any dose of Hepatitis B vaccine?” Those who answered “YES” were further asked the following to determine complete vaccination status: IF YES, how many doses have you received so far? The forgoing question has the following response scale: 1 dose, 2 doses, 3 or more doses. HCWs who had received at least 3 doses were classified as those who have received complete hepatitis B vaccination. Explanatory variables under investigation included gender, occupation, and years of training.

### Data analysis

Data was analyzed using STATA version 14 software. Each of the selected countries was analyzed separately. Analysis was mainly in two stages. The first stage involved descriptive analysis during which weighted estimates of the outcome and associated 95% confidence intervals were provided. Weighting was done due to the unequal probability of inclusion of cases from certain subgroups in the DHS sample. All included health care facilities were selected from a sample frame of listed health care facilities stratified by facility type, regional location, and management type (i.e., public vs. private facilities). Hospital facilities are usually oversampled because of their relative numbers in each country. Sample weight is a function of the selection probability of health facility and the selection probability within the subgroup of the individual. Sample weights were normalized and estimated in six decimals but often presented in the DHS data without the decimals. Therefore, prior to applying the sample weights during analysis, the sample weight for each case was divided by 1,000,000. All weighting adjustments were done in STATA-14. Thus, sample weights were applied to adjust for representation and ensure accurate estimates of the outcome. Given that the outcome variable is binary, we used binary logistic regression to build the multivariable model showing estimates of the predictors of complete dosing of hepatitis B vaccination among healthcare providers. In the multivariable model, we reported odds ratios and its associated 95% confidence intervals.

### Data availability

Data used for the present study is freely available after online request at https://dhsprogram.com/data/dataset_admin/index.cfm.

### Ethical consideration

Ethical clearance to undertake the survey was granted by the respective national authorizing bodies. Informed consent was obtained from the health care providers prior to being interviewed. Owing to the use of secondary data, no further consents were sought.

## Results

### Demographic characteristics

The proportion of HCWs who received the required doses of hepatitis B vaccine in Afghanistan, Haiti, Malawi, Nepal, and Senegal were 69.1%, 11.3%, 15.4%, 46.5%, and 19.6%, respectively. There were more males than female HCWs in Afghanistan and Malawi. Haiti, Nepal, and Senegal datasets contained more females than male HCWs. In the Afghanistan sample, many of the HCWs were medical doctors. There were more nurses and midwives than other HCWs in Haiti and Senegal as shown in Table 1.

**Table 1:** Summary statistics of the study variables (estimated prevalence and sample characteristics)

	Afghanistan	Haiti	Malawi	Nepal	Senegal
	n (%); 95% CI	n (%); 95% CI	n (%); 95% CI	n (%); 95% CI	n (%); 95% CI
Received required doses of Hepatitis B vaccine					
No	321 (30.9); 27.4, 34.7	3955 (88.7);87.6, 89.6	2249 (84.6); 82.9, 86.1	2171 (53.5); 51.22, 55.77	1088 (80.4); 78.2, 82.4
Yes	717 (69.1); 65.3, 72.6	506 (11.3); 10.4, 12.4	411 (15.4); 13.9, 17.1	1886 (46.5); 44.23, 48.78	265 (19.6); 17.6, 21.8
Gender					
Male	580 (55.9)	1066 (23.9)	1555 (58.5)	2004 (49.4)	427 (31.56)
Female	458 (44.1)	3395 (76.1)	1105 (41.5)	2053 (50.6)	926 (68.44)
Occupational qualification					
Medical Doctors	473 (45.6)	940 (21.1)	31 (1.18)	362 (8.9)	96 (7.1)
Nurses and Midwives	313 (30.1)	2414 (54.1)	1,016 (38.23)	1588 (39.1)	669 (49.5)
Other professionals	252 (24.3)	1106 (24.8)	1612 (60.59)	2108 (51.9)	588 (43.5)
Years of education^a^	17.64 (3.88)/8–29 years	17.41 (3.12)/3–40 years	14.63 (2.41)/8–28 years	13.62 (2.87)/3–29 years	14.00 (5.17)/0–30
Weighted total	*N* = 1038.0	*N* = 4461.0	*N* = 2660.0	*N* = 4057.0	*N* = 1353

^a^M (SD)/Min–Max

### Correlates of the required doses of hepatitis B among healthcare workers

Among the five countries under observation, gender was significantly associated with the required doses of hepatitis B vaccination among HCWs in Haiti only (Table 2). Compared to male HCWs in Haiti, female HCWs in Haiti [AOR = 1.59, CI 1.23, 2.06] were more likely to receive the required doses of hepatitis B vaccination (Table 2). In all the five countries, the study observed that a unit increase in years of education was associated with a higher likelihood of receiving the required doses of hepatitis B vaccination (Table 2). Occupational qualification (the type of profession) was significantly associated with receiving the required doses of hepatitis B vaccine, but the category of the qualification that was protective varied from country to country. Compared to medical doctors, nurses and midwives [AOR = 5.95, CI 2.23, 15.88], and other professionals [AOR = 8.57, CI 2.92, 25.09] were more likely to receive the required doses of Hepatitis B vaccine in Afghanistan. In Haiti, nurses and midwives [AOR = 0.22, CI 0.16, 0.31], and other professionals [AOR = 0.19, CI 0.12, 0.29] were less likely to receive the required doses of Hepatitis B vaccine compared to medical doctors. Compared to medical doctors, other professionals [AOR = 0.30, CI 0.11, 0.76] were less likely to receive the required doses of hepatitis B vaccine in Malawi. Compared to medical doctors, nurses and midwives [AOR = 0.29, CI 0.20, 0.43] and other professionals [AOR = 0.22, CI 0.15, 0.31] were less likely to receive the required doses of hepatitis B vaccination in Nepal. In Senegal, nurses and midwives [AOR = 0.23, CI 0.12, 0.57] were less likely to receive the required doses of hepatitis B vaccination compared to medical doctors.

**Table 2:** Gender, occupational qualification, and years of education regressed on required hepatitis B doses among healthcare workers

Variable name	Afghanistan		Haiti		Malawi		Nepal		Senegal	
	OR [95% CI]	AOR [95% CI]	OR [95% CI]	AOR [95% CI]	OR [95% CI]	AOR [95% CI]	OR [95% CI]	AOR [95% CI]	OR [95% CI]	AOR [95% CI]
Gender										
Male	1	1	1	1	1	1	1	1	1	1
Female	1.05 [0.74, 1.48]	1.24 [0.79, 1.94]	0.62 [0.49, 0.77]	1.59 [1.23, 2.06]	1.53 [1.20, 1.93]	1.04 [0.81, 1.36]	0.83 [0.69, 1.00]	0.82 [0.60, 1.12]	0.97 [0.58, 1.62]	1.63 [0.98, 2.71]
Occupational qualification										
Medical Doctors	1	1	1	1	1	1	1	1	1	1
Nurses and Midwives	0.96 [0.65, 1.40]	5.95 [2.23, 15.88]	0.22 [0.18, 0.27]	0.22 [0.16, 0.32]	0.37 [0.18, 0.75]	0.70 [0.29, 1.66]	0.12 [0.09, 0.17]	0.29 [0.18, 0.46]	0.08 [0.03, 0.20]	0.23 [0.12, 0.57]
Other professionals	1.21 [0.76, 1.94]	8.57 [2.92, 25.09]	0.16 [0.12, 0.23]	0.19 [0.12, 0.29]	0.12 [0.06, 0.25]	0.30 [0.11, 0.76]	0.12 [0.09, 0.16]	0.22 [0.14, 0.33]	0.03 [0.01, 0.10]	0.20 [0.03, 1.52]
Years of education	1.04 [1.00, 1.09]	1.32 [1.16, 1.51]	1.21 [1.17, 1.26]	1.05 [1.004, 1.11]	1.21 [1.15, 1.28]	1.13 [1.06, 1.20]	1.20 [1.16, 1.25]	1.12 [1.07, 1.18]	1.27 [1.17, 1.38]	1.18 [1.02, 1.37]

## Discussion

This study found that the estimated national prevalence of required hepatitis B vaccination coverage among HCWs in Afghanistan, Haiti, Malawi, Nepal, and Senegal were 69.1%, 11.3%, 15.4%, 46.5%, and 19.6%, respectively. The study also found gender to be significantly associated with hepatitis B vaccination among HCWs in Haiti. Years of education was protective for hepatitis B vaccination among HCWs in all five countries. Similarly, occupational qualification (type of profession) was significantly associated with hepatitis B vaccination among HCWs, with the protective occupational qualification varying from country to country.

The differences in diagnostic techniques and immunization protocols in these countries may be contributing factors to the discrepancies in the uptake of hepatitis B vaccination among the HCWs [44]. The low coverage in some countries may be due to the cost and unavailability of vaccination [13, 15], while the high coverage in some countries such as Afghanistan is likely due to local and national policies that ensure the availability of free hepatitis B vaccine for the HCWs [3, 15, 23]. The study findings of low prevalence (11.3%) in Haiti was similar to results from Cameroon (11.4%) [45] and Burkina Faso (10.9%) [23]. Afghanistan’s relatively high coverage (69.1%) compares to that of China (60%) [15], United States of America (63.4%) [46], and Libya (62.1%) [23]. Haiti’s low coverage is not surprising because hepatitis B vaccine was only introduced into their national routine immunization schedule in 2012 [36, 37].

Afghanistan, for example, has a robust national hepatitis B immunization strategy which targets mandatory vaccination at birth, 6 weeks, 10 weeks and 14 weeks [47]. Nevertheless, this childhood hepatitis B vaccination strategy does not explain the high vaccination prevalence in adults. A recent cross-sectional survey among 502 health workers in Kabul, the Afghan Capital, found that only 77.45% had undergone screening for HBV, and 56.37% had received at least one dose of the hepatitis B vaccine [48]. Given that the forgoing results is from a study conducted in one locality, the result from our analysis is complementary and has an added advantage of the possibility of generalizability because of the nationwide representativeness of our study sample.

The study found significant association between hepatitis B vaccination status and gender, healthcare occupational qualification (type of profession) and years of education. While in general, female HCWs were more likely to receive the required doses of the hepatitis B vaccine, gender was not a statistically significant predictor in Afghanistan, Malawi, and Nepal. In Haiti and Senegal, the study found that females were more likely than male HCWs to have had a complete dose of the hepatitis B vaccine. This could be explained by the observation that, females have greater risk perception than males [10] and this could possibly be due to the high health-seeking behaviours among women in general [11]. Our result concurs with findings in similar studies conducted in Ethiopia [3, 10, 11]. Therefore, contextual gender-specific, friendly interventions can be devised to improve the uptake of hepatitis B vaccination among all HCWs.

In this study, there was significant association between occupational qualification (type of profession) and vaccination status. Except in Afghanistan and Malawi (nurses and midwives only), where other healthcare professionals and nurses were more likely than medical doctors to have received the required doses of hepatitis B vaccine, nurses and midwives and other professionals were less likely than doctors to have received the required dose of hepatitis B vaccine. This concurs with other studies that found medical doctors to have higher vaccination coverage in general than nurses and other professional groups [23, 24, 49]. This underlines the importance of targeted interventions towards all HCWs to be encouraged to vaccinate against hepatitis B especially among nurses and midwives and other HCWs. In addition, years of education were statistically significant to vaccination status across all the five countries. This concurs with previous studies that found significantly higher vaccination coverage among bachelor/first degree and master’s degree holders compared to their counterparts with associate degree/diploma and below [3, 49]. The study results are consistent with other studies conducted in the United States of America [46], Western Greece [50] and Nigeria [51]. This could be explained in terms of the key role played by education in promoting better awareness of the infectious disease and a higher acceptance of vaccination among highly educated HCWs [3, 52].

Based on the study findings, one would argue that policymakers in developing countries should mandatorily make hepatitis B vaccination and other important vaccinations key requirements for employment, especially among HCWs who regularly encounter bodily fluids of patients. This is practised elsewhere in countries such as France, where even students going into clinical practice require mandatory hepatitis B vaccination [53]. Nevertheless, in our knowledge, although global strategies highly encourage complete vaccination among health care providers with potential risk for exposure bodily fluids, these recommendations do not generally encourage discrimination of HCWs based on hepatitis B vaccination status [54]. In developing countries where health care providers may be inadequate, making vaccinations requisite for employment may be counterproductive. This calls for wholistic approach to improve vaccination rate in developing countries. Students being enrolled into healthcare-related programmes could be targeted to increase vaccination among future HCWs and help in protecting them during clinical training and their future healthcare practice [23]. In addition, various governments should commit financial resources towards free hepatitis B vaccination as part of incentives for frontline HCWs while incorporating it into national immunization programmes from birth for the entire population. With the varying, endemic incidence of hepatitis B in these countries, there should be key priorities in increasing screening uptakes to identify HCWs requiring vaccination and those with chronic infections who may find antiviral treatment rather beneficial [23, 55]. Lastly, with the resolution of the World Health Organization to drastically reduce hepatitis B infections, these countries should not hesitate to seek technical support and resources from the global union when the need arises.

### Strengths and limitations of the study

The study relied on nationally representative health facilities datasets, which increases the validity of generalizing the estimated results. However, the study has certain limitations. First, the survey has limited variables on socioeconomic and demographic characteristics. As a result, the study relied on just a few variables to build the logistic regression models. Second, the associations between the outcome variable and the explanatory variables cannot be interpreted as causal given that the datasets are from population-based surveillance surveys. Lastly, the study periods of the surveys vary from one country to another. Given that national and global policy changes between survey times may affect vaccination rates, any observed inter-country variation should be interpreted in context of the differential time points for data collection.

## Conclusion

The study findings provide better insights to policymakers in the five countries to understand the prevalence of hepatitis B vaccination among HCWs by unravelling the geo-clinicodemographic factors that predict its uptake. We found the uptake of complete hepatitis B vaccination among HCWs in Afghanistan, Haiti, Malawi, Nepal, and Senegal to be 69.1%, 11.3%, 15.4%, 46.5%, and 17.6%, respectively. Gender, occupational qualification, and years of education were significant correlates of receiving the required doses of hepatitis B among HCWs. Policymakers in these developing countries should intensify education campaigns, screening uptakes to identify HCWs requiring vaccination, consider making hepatitis B vaccination a compulsory and key requirement for employment following stakeholder consultations, and support the antiviral treatment of HCWs with chronic infections. Ultimately, this will reduce the infection rate among the general population.

## Data Availability

Data used for the present study is freely available after online request at https://dhsprogram.com/data/dataset_admin/index.cfm. The dataset supporting the conclusions of this article is included within the article.

## Abbreviations

AOR: Adjusted odd ratio
AMR: Antimicrobial resistance
HBV: Hepatitis B virus
CDC: Centre for Disease Control and Prevention
CI: Confidence interval
DHS: Demographic and Health Survey
HCW: Health care worker
SPA: Service Provision Assessment
WHO: World Health Organization
